# Simulation of Soil Temperature under Plateau Zokor’s (*Eospalax baileyi*) Disturbance in the Qinghai Lake Watershed, Northeast Qinghai–Tibet Plateau

**DOI:** 10.3390/ani13172703

**Published:** 2023-08-24

**Authors:** Ting Xie, Yu-Jun Ma

**Affiliations:** School of Geography and Planning, Sun Yat-sen University, Guangzhou 510275, China; xiet55@mail2.sysu.edu.cn

**Keywords:** soil temperature, plateau zokor, simultaneous heat and water model, soil properties, vegetation characteristics

## Abstract

**Simple Summary:**

The plateau zokor (*Eospalax baileyi*) is one of the main native soil mammals on the Qinghai–Tibet Plateau, and plays a key role in the terrestrial ecosystem there. Its disturbance will alter the soil properties and vegetation characteristics, and further, distinctly affect soil temperature. This study analyzed the soil temperature variation in three land surface types (grassland, mound, and bald patch) under the plateau zokor’s disturbance from October 2018 to July 2020, and investigated the effect of environmental parameters on soil temperature change in the Qinghai Lake watershed. The results showed that: (1) The daily range of soil temperature was mound > bald patch > grassland, which became smaller as the depth increased. The appearance time of the maximum and minimum soil temperature at most depths showed the following order: mound, bald patch, then grassland. (2) The SHAW (simultaneous heat and water) model was applicable for the simulation of soil temperature under the plateau zokor’s disturbance, especially during the growing season. (3) The influence of vegetation characteristics on soil temperature change was relatively smaller compared with soil properties after the disturbance of plateau zokor.

**Abstract:**

The soil temperature is a key factor affecting the fragile terrestrial ecosystems on the Qinghai–Tibet Plateau, and has been remarkably altered by the soil mammal’s disturbance. This study first analyzed the soil temperature variation in grassland, mound, and bald patch under the disturbance of plateau zokor (*Eospalax baileyi*) from October 2018 to July 2020 in the Qinghai Lake watershed. Then, the SHAW (simultaneous heat and water) model was used to simulate the soil temperature change of three land surface types, and the sensitivity of soil temperature to environmental parameters before and after the disturbance was explored. The results showed the following: (1) The daily range of soil temperature was mound > bald patch > grassland, which became smaller as the depth increased, due to the co-influence of vegetation coverage and soil bulk density. There was an obvious hysteresis of soil heat transfer for grassland, as compared with mound and bald patch, especially at 5 and 15 cm depths. (2) The SHAW model was applicable for the simulation of soil temperature under the plateau zokor’s disturbance, especially during the growing season, and had better simulation accuracy for deep soil. (3) Air-entry potential and pore-size distribution index obviously affected soil temperature change, because of the change in root system and soil pores under the plateau zokor’s disturbance. With the evolution of disturbance process, the response of soil temperature to the leaf area index weakened gradually, owing to the different duration of disturbance and restoration. In general, the plateau zokor’s disturbance alters the soil properties and vegetation characteristics, and further, distinctly affects heat transfer and soil temperature.

## 1. Introduction

The Qinghai–Tibet Plateau is the highest plateau in the world, with an average altitude of more than 4000 m and an area of nearly 2.58 million square kilometers; therefore, it has the title of “the world’s third pole” [1,2]. The unique geographical location and topographic features make the Qinghai–Tibet Plateau more susceptible to regional and even global atmospheric circulation, becoming the “initiator”, or a more sensitive area to climate change [3,4,5]. On the other hand, the Qinghai–Tibet Plateau affects the development of glaciers, permafrost, lakes, etc., and plays a key role in the evolution of the regional ecosystem [6].

The high altitude, cold, and drought characteristics of the Qinghai–Tibet Plateau make the regional ecosystem very fragile [7]. First of all, due to climate warming, the acceleration of glacial melting and permafrost degradation has increased the thickness of the active layer, affecting the heat transfer between the atmosphere and the soil, which in turn affects the ecosystems of the Qinghai–Tibet Plateau [8,9]. Secondly, human activities (e.g., excessive grazing and expansion of infrastructure construction) have aggravated the degradation of ecosystem, affecting ecological security to a certain extent [10,11,12]. Changes in soil properties, vegetation types, and coverage are the main manifestations of alpine meadow degradation [13]; meadows provide suitable environmental conditions for the survival and reproduction of small soil mammals such as the plateau zokor (*Eospalax baileyi*) and plateau pika (*Plateau zokor*) [14]. They, conversely, alter the surface micro-landscapes by gnawing grass, digging tunnels, excreting feces, and other disturbance behaviors, and affect the soil physical structure, chemical properties, and hydrological processes, thus become an important influencing factor in the process of ecosystem change on the Qinghai–Tibet Plateau [15,16,17,18].

The soil temperature change will vary the energy transmission between the ground and the atmosphere, which in turn influences regional atmospheric circulation and even climate change, thus affecting the regional ecosystem [19,20]. For example, the increase in soil temperature will accelerate the permafrost degradation, which in turn promotes the transfer of carbon from the soil to the atmosphere and enhances climate warming on the Qinghai–Tibet Plateau [8,21,22]. Due to the wide distribution of soil mammals, their disturbance will change the surface landscape and soil properties, cause variation in soil heat transfer, and affect the climate change of the Qinghai–Tibet Plateau.

The environmental conditions of the Qinghai–Tibet Plateau are relatively harsh, making it difficult to carry out long-term experiments. The land surface process model can effectively simulate the energy transfer between the ground and the atmosphere, so it has become the main tool for the study of heat exchange and parameter sensitivity analysis in this area. Many models, such as Community Land Model (CLM) [23], Geophysical Institute Permafrost Laboratory 2.0 (GIPL2) [11], and Coupled Heat and Mass Transfer Model for the soil–plant–atmosphere system (CoupModel) [24], can effectively simulate soil heat change in cold regions. However, there are still some problems, such as complex model structures and difficulty in obtaining some input parameters through experiments. The Simultaneous Heat and Water (SHAW) model is a one-dimensional model that can well simulate the heat transfer between the ground and atmosphere in freezing and thawing areas [25] that is easy to operate and has convenient to obtain parameters. It has good applications in the central Qinghai–Tibet Plateau, Qilian Mountains, and other areas [26,27], but its applicability has not yet been verified under the soil mammal’s disturbance.

Therefore, using the SHAW model to explore the soil temperature change is helpful to understand the impact of soil mammal disturbance on the Qinghai–Tibet Plateau, and it provides a scientific basis for maintaining the stability of fragile ecosystems. This study mainly provides three results: (1) the variation characteristics of soil temperature for different land surface types due to plateau zokor disturbance; (2) the applicability of the SHAW model for the simulation of soil temperature under plateau zokor disturbance; (3) the sensitivity of soil temperature change to soil and vegetation parameters in the process of plateau zokor disturbance.

## 2. Materials and Methods

### 2.1. Study Area

The study area was located in the Shaliu River sub-watershed (100.26° E, 37.24° N) of the Qinghai Lake Watershed in the northeast of Qinghai–Tibet Plateau, with an altitude of 3212 m. It belongs to a high-altitude, cold and semiarid climate zone. During the observation period (17 October 2018 to 31 July 2020), the average air temperature was −0.88 °C, and relative humidity was 64.26%. The zonal soil of the study area is Cacic-orthic Aridisol according to U.S. Soil Taxonomy classification [28]. The typical native plant species are *Poa malaca* Keng, *Glaux maritima* L., and *Saussurea thoroldii* Hemsl.

### 2.2. Experiment Design

The digging behavior of the plateau zokor alters the land surface landscape: the undisturbed landscape is “grassland”, and then the soil is carried from below ground to the land surface forming a loose “mound” after excavation. Finally, under the influence of gravity, freeze–thaw, and so on, the “mound” is compacted to form a “bald patch”. Therefore, three soil profiles with a depth of 40 cm were dug in each land surface type (grassland, mound, and bald patch), and the soil bulk density was measured using the core method [29] at 0–10, 10–20, 20–30, and 30–40 cm. Soil was collected at each depth from three profiles using a spade, and mixed to produce a composite sample with a total weight of about 1 kg. Then, all the soil samples were air-dried in the laboratory and sieved to pass through a 2 mm screen. The soil texture was analyzed using the pipette method [30], and the soil organic matter content was determined using the dichromate oxidation method [31].

Three quadrats of 1 m × 1 m were selected on each of the grassland, mound, and bald patch. The number and height of dominant species in each quadrat were measured. The leaf water potential of the dominant species was measured using the WP4 dew point water potential meter (METER, Pullman, WA, USA), and their root depths were measured using the digging method, i.e., the principal root of typical native plant species was dug carefully until its end using the spade, and the vertical depth from the land surface to the root end was measured using a ruler. After cutting the plants completely and recording the number of leaves, all the leaves were placed on graph paper one by one in the laboratory, and each leaf was drawn with Image J software after vertical shooting to obtain the estimated leaf area and leaf width. After calculating the average leaf area and leaf width of a single leaf, the leaf area per plant could be obtained in combination with the number of leaves per plant. On this basis, the leaf area per plant was multiplied by the number of plants in the quadrat and then divided by the quadrat area to obtain the leaf area index.

The air temperature and relative humidity were measured with an HMP155A temperature and relative humidity probe (Vaisala, Vantaa, Finland) at a height of 2.5 m, wind speed and direction were recorded using the Windsonic sensor (Gill, Lymington, UK) placed 2 m above the ground, precipitation was measured with a TE525 tipping bucket rain gauge (Campbell Scientific, Logan, UT, USA), and solar radiation was observed using a CNR4 net radiometer (Kipp & Zonen, Delft, The Netherlands). The soil temperature and soil moisture at depths of 5, 15, 25, and 35 cm for different land surface types were observed using 5TE buried sensors (METER, Pullman, WA, USA). All observation data were automatically recorded at 10 min intervals. The growing season and the non-growing season were from May to September and from October to April of the following year, respectively [32].

### 2.3. Model Description

The SHAW model not only uses mathematical methods to describe the material and energy exchange process in detail, but also make detailed regulations for soil freezing and thawing, so as to more accurately simulate the water and heat dynamics in the process of soil freezing and thawing [33].

The coupling partial differential equation of one-dimensional vertical heat flux based on liquid convection heat transfer and steam latent heat is as follows:

(1) $$C_{s}\frac{\partial T}{\partial t}-\rho_{i}L_{f}\frac{\partial \theta_{i}}{\partial t}=\frac{\partial }{\partial z}\left[k_{s}\frac{\partial T}{\partial z}\right]-\rho_{l}c_{l}\frac{\partial q_{l}T}{\partial z}-L_{v}\left(\frac{\partial q_{v}}{\partial z}+\frac{\partial \rho_{v}}{\partial t}\right)$$

where *C_s_* is volumetric heat capacity of soil (J/kg/°C), *T* is soil temperature (°C), *t* is time (s), *ρ_i_* is ice density (kg/m^3^), *L_f_* is latent heat of fusion (33,500 J/kg), *θ_i_* is volumetric ice content (m^3^/m^3^), *z* is distance from surface (m), *k_s_* is soil thermal conductivity (W/m/K), *ρ_l_* is water density (1000 kg/m^3^), *c_l_* is specific heat capacity of water (J/m^3^/°C), *q_l_* is liquid water flux (m/s), *L_v_* is the latent heat of vaporization (J/kg), *q_v_* is water vapor flux (kg/m^2^/s), and *ρ_v_* is vapor density within the soil (kg/m^3^).

The volumetric heat capacity of soil is the sum of the volumetric heat capacity of soil components. The formula is as follows:

(2) $$C_{s\;}=\sum \rho_{j}c_{j}\theta_{j}$$

where *ρ_j_*, *c_j_*, and *θ_j_* are the density, specific heat capacity, and volumetric fraction of the *j*th soil constituent.

The model uses the De Vries theory to calculate soil thermal conductivity, and the formula is as follows:

(3) $$k_{s}=\frac{\sum m_{j}k_{j}\theta_{j}}{\sum m_{j}\theta_{j}}$$

where *m_j_*, *k_j_*, and *θj* are the weighting factor, thermal conductivity, and volumetric fraction of the *j*th soil constituent.

The operation of the SHAW model required the provision of site data, meteorological data, soil data, and vegetation data. The site data mainly referred to the latitude, longitude, altitude, slope, and aspect. Meteorological data included air temperature, relative humidity, wind speed, precipitation, and solar radiation. Soil data included hourly soil temperature and soil water content, soil texture, bulk density, pore-size distribution index, air-entry potential, saturated volumetric moisture content, saturated hydraulic conductivity. Vegetation data included plant height, leaf area index, leaf area index, and root depth. For all the required data, most were measured by ourselves (see Section 2.2), except the pore-size distribution index, air-entry potential, saturated volumetric moisture content, and saturated hydraulic conductivity, which were calculated by the model. For the operation of the SHAW model, the calibration period was from 17 October 2018 to 31 August 2019, while the verification period was from 1 September 2019 to 31 July 2020.

### 2.4. Data Analysis

All the measured data were grouped by land surface types (grassland, mound, and bald patch) and depths (5, 15, 25, and 35 cm), and differences between them were analyzed with a one-way analysis of variance. The goodness of fit (R^2^), root mean square error (RMSE), and Nash–Sutcliffe efficiency coefficient (NSE) were selected as the main indicators to evaluate the applicability of SHAW model. The R^2^ and NSE approached 1 and RMSE approached 0, indicating that the simulation efficiency was better.

The one factor at a time (OAT) method was used to analyze the sensitivity of soil temperature change to soil and vegetation parameters; that is, only one of the parameter values was changed every time the model was run [34]. In the actual operation process, the soil and vegetation parameters of the previous land surface type were sequentially replaced with the corresponding parameters of the current land surface type. The magnitude of sensitivity is expressed by relative sensitivity (RS) as follows [35]:

(4) $$RS=\left|\frac{\left[y\left(x+∆x\right)-y\left(x\right)\right]/y\left(x\right)}{∆x/x}\right|$$

where *RS* is the relative sensitivity, *x* is a parameter value, ∆*x* is the change of the parameter value, and *y*(*x*) and *y*(*x* + ∆*x*) are the simulated output value of soil temperature before and after the parameter change.

## 3. Results

### 3.1. Variation of Measured Soil Temperature

**Figure 1:**
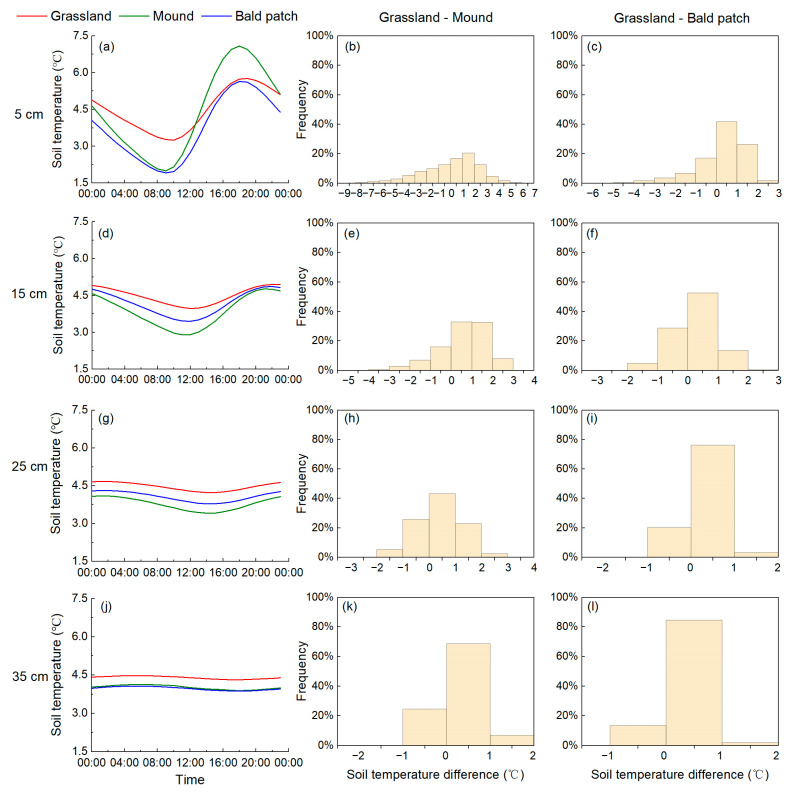
Diurnal variation of soil temperature for different land surface types in the Qinghai Lake watershed from October 2018 to July 2020. (**a**,**d**,**g**,**j**) are the diurnal variation of soil temperature for different land surface types at 5, 15, 25 and 35 cm depths, respectively. (**b**,**e**,**h**,**k**) are the soil temperature difference between grassland and mound at 5, 15, 25 and 35 cm depths, respectively. (**c**,**f**,**i**,**l**) are the soil temperature difference between grassland and bald patch at 5, 15, 25 and 35 cm depths, respectively.

From October 2018 to July 2020, the mean soil temperature of grassland was significantly higher than mound and bald patch at the 15, 25, and 35 cm depths (*p* < 0.05), but there was no significant difference at the 5 cm depth between them. For different depths, the soil temperature was significantly higher at the 5 cm depth than that at the 25 cm depth for mound (*p* < 0.05), and significantly lower at the 5 cm depth than that at 15 cm of depth for bald patch (*p* < 0.05), while the difference between the other depths was not significant for each land surface type. The soil temperature of three land surface types showed similar diurnal changes, and its variation gradually became flat with increasing depth. The soil temperature change patterns of three land surface types at the 5 and 15 cm depths were all “falling-rising-falling”, and its daily range showed the following order: mound > bald patch > grassland (Figure 1a,d). The soil temperature patterns of the three land surface types at the 25 and 35 cm depths were all “rising-falling-rising” (Figure 1g,j). The value of grassland was always the highest at these two depths, and the mound was always lower than the bald patch at the 25 cm depth, though inverse at 35 cm of depth. Generally, the variation range of soil temperature in each layer of three land surface types was mound > bald patch > grassland, which became smaller with increasing depth.

In addition, there was an obvious hysteresis of soil heat transfer as the depth deepened. The maximum soil temperature at 5 cm of depth appeared at 19:10 (Beijing time, the same below) in grassland, which was 50 and 30 min later than in mound and bald patch, respectively (Figure 1a). At the depth of 15 cm, the maximum soil temperature in the grassland appeared at 23:00, which was 70 and 40 min later than that of the mound and bald patch, respectively (Figure 1d). At the above two depths, the lowest soil temperature in the grassland appeared at 10:00 and 12:40, respectively; this was 40 min later than the mound and 30 min later than the bald patch, respectively. In general, the plateau zokor’s disturbance altered the transfer process of soil heat, and the appeared time of the maximum and minimum soil temperature at most depths showed the following order: mound, bald patch, and grassland.

Taking grassland as a reference, the range of soil temperature difference between mound and grassland, and between bald patch and grassland of each depth decreased with increasing depth, and the frequency of soil temperature difference >0 °C was higher than that of <0 °C. Among them, 0~1 °C was the concentrated range, which mainly occurred from 16:40 to 5:00 the next day. Comparing the soil temperature difference between grassland and bald patch, its difference between grassland and mound was larger, especially at 5 cm of depth, i.e., −6 to 3 °C for the former (Figure 1c) and −9 to 7 °C for the latter (Figure 1b), indicating that the plateau zokor’s disturbance evidently changed soil heat characteristics.

**Figure 2:**
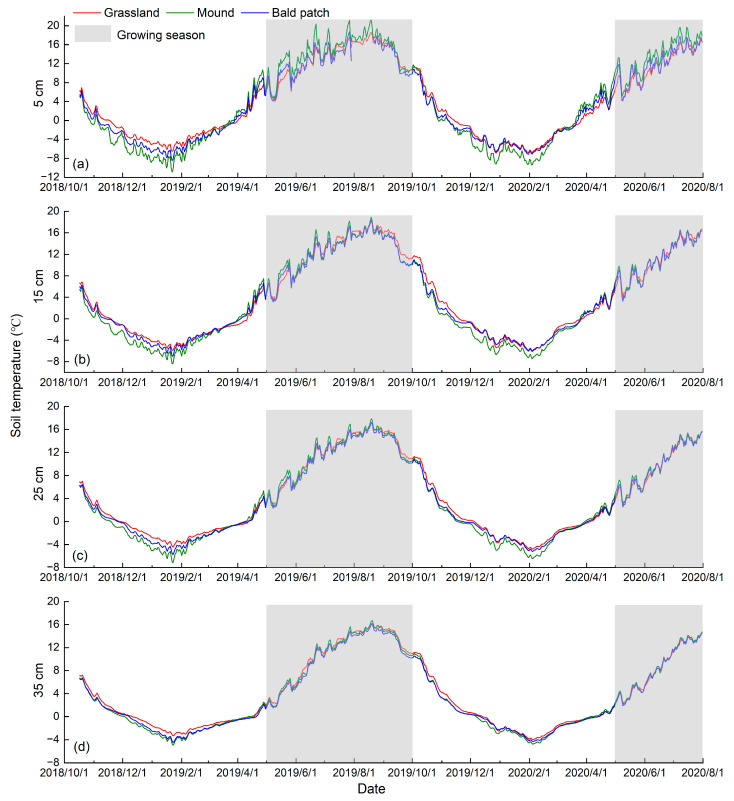
Annual variation of soil temperature for different land surface types in the Qinghai Lake watershed from October 2018 to July 2020. (**a**–**d**) are the annual variation of soil temperature for different land surface types at 5, 15, 25 and 35 cm depths, respectively.

From October 2018 to July 2020, the soil temperature at each depth of grassland, mound, and bald patch in the Qinghai Lake watershed presented a trend of approximate cosine curve (Figure 2). During the growing season, the soil temperature fluctuated greatly; while it was relatively gentle in the non-growing season. The difference between the maximum and minimum soil temperature of each depth presented as mound > bald patch > grassland, e.g., 32.09, 27.22, and 25.50 °C at the 5 cm depth, respectively. It showed that the plateau zokor’s disturbance would cause obvious changes in soil temperature, and its variation in the early stage of the disturbance process (grassland turned into mound) was higher than that in the later stage (mound compacted into bald patch).

In terms of the occurrence time of the maximum soil temperature, it was generally consistent for three land surface types. However, the occurrence time of the minimum soil temperature at different depths in the grassland was not close to each other. Therefore, the plateau zokor’s disturbance had no distinct influence on the appearance time of the maximum soil temperature, but had a remarkable effect on the appearance of the minimum value.

### 3.2. Simulation of Soil Temperature

**Figure 3:**
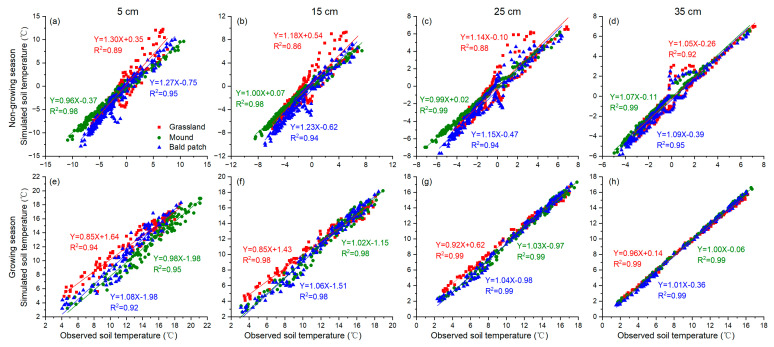
Comparison of simulated and observed soil temperature at different depths of different land surface types in the Qinghai Lake watershed from October 2018 to August 2019. (**a**–**d**) are the regression between simulated and observed soil temperature during non-growing season at 5, 15, 25 and 35 cm depths, respectively. (**e**–**h**) are the regression between simulated and observed soil temperature during growing season at 5, 15, 25 and 35 cm depths, respectively.

During the calibration period, the simulation results of soil temperature for three land surface types were generally consistent with the observation data, and as the depths increased, the simulation efficiency was better (Figure 3). For R^2^, RMSE, and NSE, all the evaluation indexes of the mound were better than those of the grassland at all depths, except the RMSE at 5 cm depth (Table 1). The RMSE and NSE of each layer of the bald patch were similar to those of the grassland. This indicated that the SHAW model had high simulation accuracy for the soil temperature in the Qinghai Lake watershed, and it could better simulate the influence of mounds formed by plateau zokor’s disturbance on the deep soil temperature.

**Figure 4:**
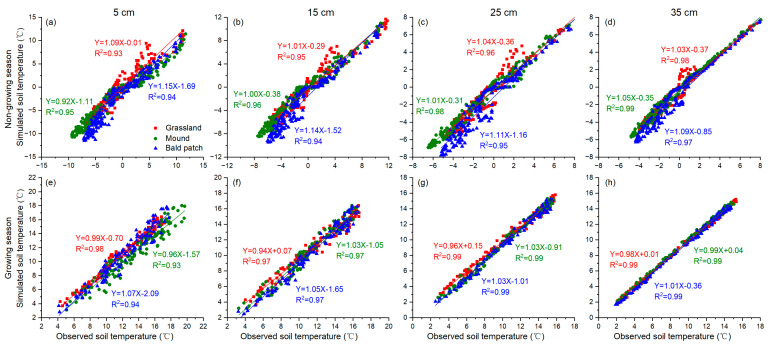
Comparison of simulated and observed soil temperature at different depths of different land surface types in the Qinghai Lake watershed from September 2019 to July 2020. (**a**–**d**) are the regression between simulated and observed soil temperature during non-growing season at 5, 15, 25 and 35 cm depths, respectively. (**e**–**h**) are the regression between simulated and observed soil temperature during growing season at 5, 15, 25 and 35 cm depths, respectively.

During the verification period, the simulated curve of the soil temperature of grassland, mound, and bald patch in the Qinghai Lake watershed were in good agreement with the observed curve, indicating that the SHAW model was applicable for this area (Figure 4). In addition, the R^2^ of grassland, mound, and bald patch were all greater than 0.97 and all the NSE values were above 0.92 (Table 1). Both the R^2^ and the NSE increased with the depth, while the RMSE decreased sequentially from the surface layer to the deep layer, indicating that the SHAW model had better simulation accuracy for deep soil.

### 3.3. Sensitivity of Soil Temperature Change

**Figure 5:**
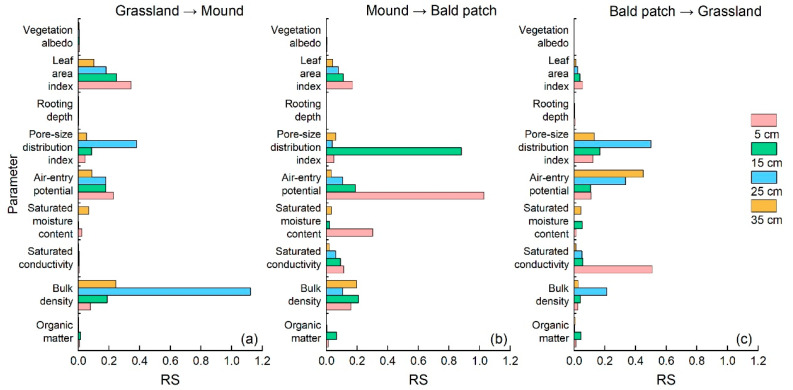
Relative sensitivity (RS) of soil temperature change to soil and vegetation parameters. (**a**–**c**) are the relative sensitivity during the disturbance processes from grassland to mound, from mound to bald patch, and from bald patch to grassland, respectively.

In turn, the values of different environmental parameters of grassland were replaced with the corresponding values of mound. The steps for the evolution of mound to bald patch and the restoration of bald patch to grassland were also the same. Through the above operations, the sensitivity of soil temperature to the changes in soil and vegetation parameters during three disturbance processes was determined and is shown in Figure 5.

In general, air-entry potential and pore-size distribution index were the two parameters that had obvious influence on the change of soil temperature. When grassland was disturbed into mound, soil temperature had a high RS to the change of soil bulk density at the 25 cm depth (Figure 5a), and the responses of soil temperature to air-entry potential and leaf area index weakened with increasing depth. During the compaction of the mound to the bald patch, the RS of soil temperature to air-entry potential at 5 cm depth and pore-size distribution index at 15 cm depth were relatively high (Figure 5b). In the process of restoring bald patch to grassland, soil temperature had a high RS for saturated conductivity at 5 cm of depth, pore-size distribution index at 25 cm of depth, and air-entry potential at 35 cm of depth (Figure 5c). Comparing three disturbance processes, it can be found that the RS of soil temperature in each layer to the change of leaf area index decreased with the increase in depth. With the evolution of the disturbance process, the effect of leaf area index changes weakened gradually. During the mound compaction to bald patch, the sensitivity of soil temperature at the 5 cm depth to saturated moisture content was higher than that in the other two disturbance stages.

## 4. Discussion

### 4.1. Reasons for Soil Temperature Difference under Disturbance

Our measurements found that both the daily and annual ranges of soil temperature for grassland were lower than that of mound and bald patch at the corresponding depths (Figure 1 and Figure 2). Previous studies showed that the albedo of grassland was significantly higher than that of mound after the plateau pika’s disturbance [36], which means that more solar radiation will be reflected by grassland. Moreover, dense vegetation could effectively reduce the impact of solar radiation on soil heat transfer, thereby reducing fluctuations in soil temperature [37]. The excavation of plateau zokor destroyed the land surface vegetation, and the plant coverage after the plateau zokor’s disturbance was lower significantly than that of grassland [38]. Therefore, the fluctuation of soil temperature for mound and bald patch was remarkably larger than that of grassland, especially at the 5 cm depth.

The plateau zokor’s disturbance also changed soil properties, which affected the soil temperature in turn. It dug tunnels underground and moved the deep soil to the land surface, which changed soil bulk density and porosity, and further affected the transmission of soil heat [39,40]. The variation of soil temperature for mound at each depth was greater than that of bald patch, and the difference between the mound and grassland was also larger than that between the bald patch and grassland. The reason was that there was a difference in soil bulk density between loose mound and compacted bald patch. The bulk density of the former at the 5 and 15 cm depths were 960 and 1150 kg/m^3^, respectively; while the latter were 1220 and 1230 kg/m^3^, respectively. At the 25 and 35 cm depths, the differences in soil bulk density between mound and bald patch were -10 and 30 kg/m^3^, respectively. The increase in soil bulk density reduced soil temperature [41], so the shallow soil temperature variation of the mound was higher than that of the bald patch.

### 4.2. Applicability of SHAW Model under Disturbance

In general, the SHAW model had high simulation accuracy of soil temperature for all three land surface types under the plateau zokor’s disturbance in the Qinghai Lake watershed (Table 1). For different depths, the SHAW model had better simulation accuracy for deep soil. This was because the plant roots were concentrated at a depth of 0–20 cm, and the plateau zokor’s disturbance mainly alters the surface landscape and shallow soil properties.

The simulation deviation of soil temperature by the SHAW model was relatively large during the non-growing season (Figure 3 and Figure 4). There were three main reasons: Firstly, vegetation plays a key role in surface albedo changes in the eastern Qinghai–Tibet Plateau [42]. The digging behavior of the plateau zokor changed the vegetation type and its growth conditions, which in turn affected the albedo continuously. However, the albedo used by the SHAW model was only an average value and did not change with time, so it could not accurately reflect the dynamic impact of albedo changes on soil temperature. Secondly, the subsurface lateral flow was an important heat transfer process in the soil [43,44], and the convective heat transfer also had a greater contribution in the unfrozen soil [45]. These two factors had a certain influence on the soil temperature change, especially at the shallow layer. However, the SHAW model, as a one-dimensional vertical model, did not include these two aspects in the thermal simulation. Thirdly, the model simply assumed a fixed value of soil components such as particles, ice, and unfrozen water to calculate soil thermal conductivity [27]. However, with the change in time and depth, these constituents will vary obviously, which has not been sufficiently considered in the model.

### 4.3. Effects of Soil and Vegetation Parameters on Soil Temperature

Our study showed that air-entry potential and pore-size distribution index distinctly affected soil temperature under the plateau zokor’s disturbance (Figure 5). Low-density fine roots would block the soil pores and increase the air-entry potential, while thick roots would increase the development of large pores and reduce the air-entry potential [46,47]. After the plateau zokor’s disturbance, the change in root system and soil pores affected soil ventilation and water permeability [48,49], thereby altering the soil heat transfer. We found that for three land surface types, the influence of air-entry potential on soil temperature increased first and then decreased at the 5 and 15 cm depths. Additionally, a previous study pointed out that the caves dug by soil animals were vertical pores [50]. The tunnels excavated by plateau zokors could also be regarded as large pores, with a depth of 1.6 and 19.8 cm [51], which will affect the soil thermal conductivity in the vertical direction. Therefore, the sensitivity of soil temperature to air-entry potential during the restoration of bald patch to grassland was opposite to the other two processes.

The soil thermal conductivity increased with the increase in soil compactness [52]. In the process of downward heat transfer, the vegetation cover prevented energy loss [53]. The mound soil was very loose, and its surface had little vegetation; therefore, the downward transfer of energy was slower. Our research also showed that the importance of leaf area index for soil temperature gradually decreased with the depth and the disturbance process. The heat in soil mainly came from land surface radiation; therefore, the variation in soil temperature will decrease with the increase in depth. During the whole disturbance process, the moved soil by the plateau zokor covered the plant for a short time, and the land surface characteristics varied quickly. In contrast, the restoration of vegetation for mound and bald patch will take a long time [36], which will weaken the impact of leaf area index on soil temperature variation.

## 5. Conclusions

The plateau zokor’s disturbance alters the soil properties and vegetation characteristics, and further affect heat transfer and soil temperature. This study seeks to investigate the variation in soil temperature and its key driving factors under plateau zokor’s disturbance, by combining field measurements and the SHAW model.

From October 2018 to July 2020, the diurnal variation of soil temperature for grassland, mound, and bald patch were similar, and there was a hysteresis in the downward transfer of soil heat. The daily range of soil temperature was mound > bald patch > grassland, which became smaller with increasing depth. The SHAW model was generally ideal for the simulation of soil temperature on grassland, mound, and bald patch, especially in the growing season. Soil temperature was greatly affected by the air-entry potential and pore-size distribution index. In addition, it also had relatively high sensitivity to soil bulk density, saturated moisture content, saturated conductivity, and leaf area index. When soil homogeneity was high, soil temperature was most sensitive to soil bulk density changes; when soil was disturbed, the effect of air-entry potential and pore-size distribution index were higher than soil bulk density. With the evolution of disturbance process, the influence of leaf area index change on soil temperature weakened gradually.

## Figures and Tables

**Table 1:** Evaluation of simulated results at different land surface types in Qinghai Lake watershed.

Land Surface Type	Index	Calibration Period				Validation Period			
		5 cm	15 cm	25 cm	35 cm	5 cm	15 cm	25 cm	35 cm
Grassland	R^2^	0.960	0.968	0.982	0.991	0.974	0.984	0.988	0.993
	RMSE	1.600	1.336	0.935	0.639	1.252	0.987	0.773	0.573
	NSE	0.958	0.968	0.980	0.989	0.971	0.981	0.985	0.991
Mound	R^2^	0.991	0.994	0.997	0.998	0.983	0.987	0.994	0.997
	RMSE	1.646	0.780	0.531	0.321	1.902	0.969	0.665	0.463
	NSE	0.971	0.991	0.995	0.998	0.953	0.983	0.990	0.994
Bald ground	R^2^	0.974	0.983	0.989	0.994	0.971	0.976	0.981	0.986
	RMSE	1.880	1.409	0.969	0.642	2.342	2.116	1.779	1.333
	NSE	0.941	0.964	0.980	0.989	0.949	0.922	0.932	0.955

## Data Availability

Primary data used in this paper are available from the authors upon request (mayujun3@mail.sysu.edu.cn).
